# Lord of the Ring technique for capsular tension ring in subluxated cataract surgery

**DOI:** 10.1038/s41433-024-02988-4

**Published:** 2024-03-07

**Authors:** Pei-Chang Wu

**Affiliations:** Department of Ophthalmology, Kaohsiung Chang Gung Memorial Hospital and Chang Gung University College of Medicine, Kaohsiung, Taiwan

**Keywords:** Outcomes research, Medical research

**Fig. 1:**
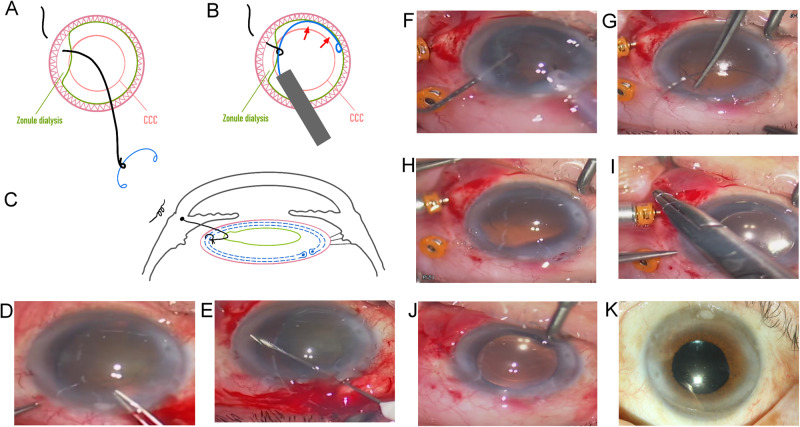
Lord of Ring technique for capsular tension ring (CTR). **A** A movable knot is tied by 10-0 prolene string in the CTR shift. **B** The CTR is slowly released into bag. **C** The other end of string is fixed to the sclera. The movable knot is moved to a good location. Moderate tension is maintained to hold the CTR. Lateral view of the string holding the ring in the zonule dialysis area. **D** A case of traumatic cataract with temporal 180° zonular dialysis (video). Circular capsulorhexis was performed gently. **E** A 10-0 string needle was inserted from the temporal sclera away limbus 2 mm into anterior chamber, and pulled out by a 27-gauge needle. **F** Phacoemulsification and gentle cortical removal can be performed, or after CTR insertion. **G** = **A**. **H** = **B**. **I** IOL was implanted and procedure in (**C**) is carried out. **J** A well-centered IOL. **K** After 3 months, vision improved to 20/40.

## Video description

A case of traumatic cataract with temporal 180° zonular dialysis received surgery with the Lord of the Ring technique.

## Supplementary information

Lord of tension ring

## Data Availability

The data are available from the corresponding author on reasonable request.

